# Impact of Stenting on PDA Length, Curvature, and Pulsatile Deformations Based on CT Assessment

**DOI:** 10.1016/j.jscai.2023.101134

**Published:** 2023-09-16

**Authors:** Christopher P. Cheng, Johan Bondesson, Sanjeet Hegde, Maria T. Acuero, Howaida G. El-Said

**Affiliations:** a Division of Vascular Surgery, Department of Surgery, Stanford University, Stanford, California; b Division of Pediatric Cardiology, Rady Children’s Hospital, UC San Diego, San Diego, California

**Keywords:** device durability, device selection, ductal-dependent circulation, patent ductus arteriosus, preoperative planning, stenting

## Abstract

**Background:**

We sought to investigate the impact of stenting on native patent ductus arteriosus (PDA) length, curvature, and pulsatile deformations in patients with ductal-dependent pulmonary circulations.

**Methods:**

Patients with PDA stents who received contrast-enhanced 3-dimensional computed tomography with a view of the PDA, thoracic aorta, and pulmonary arteries were retrospectively included in this study. Geometric models of the prestented and poststented PDA were constructed from the computed tomography images, and PDA arclength, curvature, and pulsatile deformations were quantified.

**Results:**

A total of 12 patients with cyanotic congenital heart disease were included, 10 of whom received 1 stent in the PDA and 2 received multiple overlapping stents. From prestenting to poststenting, the PDA shortened by 26 ± 18% (*P* = .004) and decreased in mean and peak curvature by 60 ± 21% and 68 ± 15%, respectively (both *P* < .001). Pulsatile deformations varied highly for the native PDA, stented PDA, and stents themselves.

**Conclusions:**

The shortening and straightening of the PDA after stenting are significant and substantial, and their quantitative characterization will enable interventionalists to select stent lengths that span the entire PDA without encroaching on the aortic or pulmonary artery, which could cause hemodynamic interference, stent kink, and fatigue. Pulsatile PDA deformations can be used to design and evaluate devices tailored to congenital heart disease in neonates.

## Highlights

• Quantification of PDA geometry and deformation done prestenting and poststenting.
• PDA was shortened and straightened after the intervention compared to before.
• Can be refined into tools for clinicians to select stent size and design.
• Data can be used to improve stent design for conformability and durability.

## Central Illustration

**Figure:**
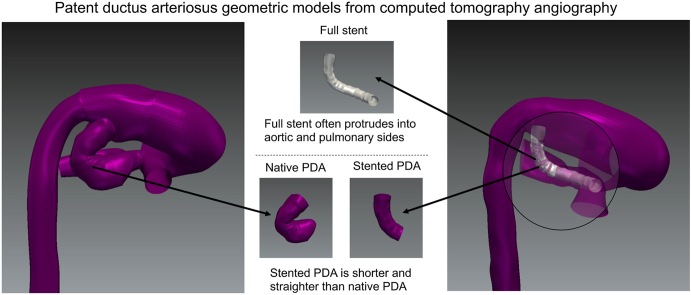

## Introduction

Annually, approximately 7200 babies in the United States are born with critical congenital heart defects severe enough to significantly lower oxygenation in the blood and cause cyanosis, prompting intervention during the first year of life.^1^, ^2^, ^3^, ^4^ For patients with ductal-dependent pulmonary circulation, the patent ductus arteriosus (PDA) must remain open to support the pulmonary circulation.^5^ Since the PDA naturally closes off shortly after birth, a stent can be placed in the PDA to maintain a flow conduit between the 2 circulations.^6^, ^7^, ^8^ Catheter-based ductus stenting, currently done off-label with adult coronary artery stents, has been highlighted as the preferred option to surgical intervention (modified Blalock-Taussig-Thomas shunt) due to comparable primary outcomes and more beneficial pulmonary artery remodeling and shorter intensive care unit stays.^5^^,^^9^

While stents implanted in the PDA are critical for supporting life in neonates, the impact of stenting on PDA morphology and pulsatile deformations has not been well characterized. Perhaps most urgently, selection of PDA stent length continues to be a challenge as it is difficult to predict the length of the PDA after it has been straightened with a stent or stiff guidewire; hence, characterizing PDA geometry prestenting and poststenting may help address this important concern. Further, there are no published data on the pulsatile deformations of native or stented PDA, and as this has been shown to be an important factor for stent durability in other vessels,^10^, ^11^, ^12^, ^13^ it was included in the study. In this context, pulsatile deformations were defined as any geometric change of the PDA during the cardiac cycle caused by pressurization of the vessel, movement of neighboring anatomy (such as the heart, aorta, or pulmonary arteries), or a combination of these. These deformations not only directly affect the mechanical durability of implanted stents already being utilized, but are critical for optimizing future stent designs dedicated to the PDA. Finally, better understanding of the pulsatile deformation of PDA could improve characterization and stratification of ductal-dependent congenital heart diseases, simulations of congenital heart hemodynamics and interventions, and prediction of prognosis of interventions.

The purpose of this study was to utilize nongated and cardiac-gated computed tomography (CT) images of neonates before and after PDA stenting to quantify the impact of stenting on PDA length and curvature as well as characterize native and stented PDA pulsatile deformations.

## Methods

### Patient recruitment and medical imaging

Patients implanted with stents in the PDA from October 2017 through April 2022 were recruited retrospectively from Rady Children’s Hospital (University of California, San Diego, California) and Nationwide Children’s Hospital (Ohio State University, Columbus, Ohio). Medical images were deidentified and data use complied with institutional review boards and data use agreements. For inclusion, 3-dimensional (3D) chest CT needed to be contrast-enhanced and include the PDA as well as the thoracic aorta and pulmonary arteries. CT images could be nongated or cardiac-gated with only diastole resolved for characterizing morphology and cardiac-gated with diastole and systole resolved for characterization of pulsatile deformation.

### Geometric modeling

The open-source SimVascular software suite (Open Source Medical Software Corporation) was used to build 3D geometric models from CT images, and quantification of geometries and deformation was processed with custom MATLAB (MathWorks) software.^14^ For static morphology analysis, a single diastolic frame from nongated or gated CT was needed. For cardiac pulsatility-induced deformation analysis, both diastolic and systolic frames, either predetermined by the CT scanner and electrocardiogram readings or chosen manually, were needed to compare geometries to quantify changes.

**Figure 1:**
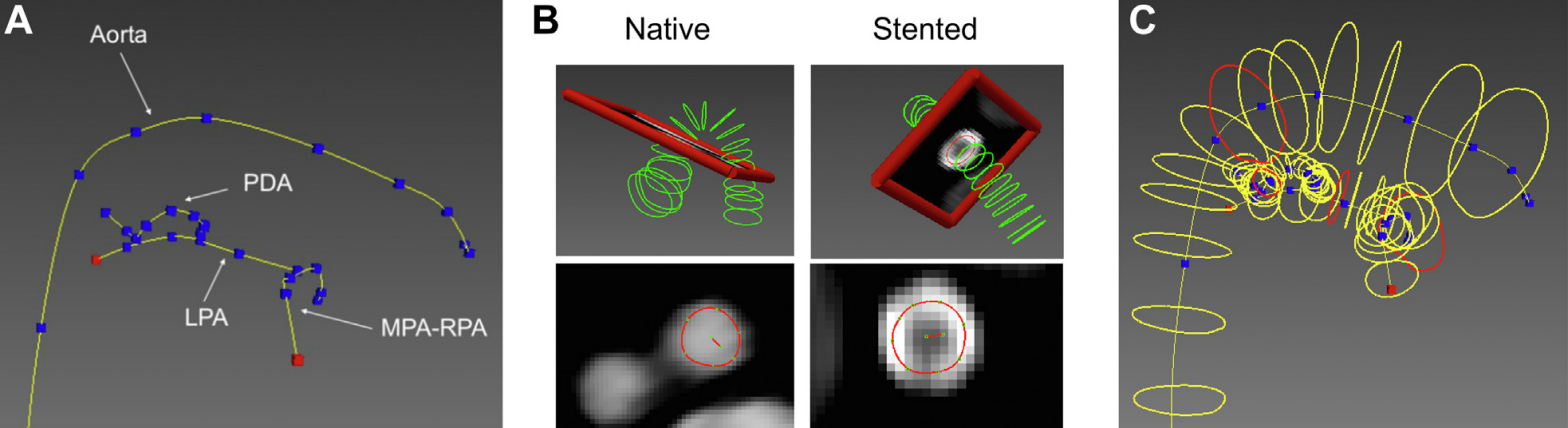
**Geometric modeling of the aorta, pulmonary arteries, and patent ductus arteriosus.** (**A**) Manual centerline path selection of the aorta, left pulmonary artery (LPA), main pulmonary artery continuing to the right pulmonary artery (MPA-RPA), and the patent ductus arteriosus (PDA) connecting the aorta to the LPA. (**B**) Cross-sectional contour segmentation of the native and stented PDA. (**C**) Contours of the aorta, pulmonary arteries, and PDA.

After identification of cardiac frames (diastole or diastole + systole), 3D geometric modeling was performed for the aorta, pulmonary arteries, and the PDA (Figure 1). The process started with hand-picking centerline points for all relevant vessels and fitting them with cubic splines, with the primary strategy of achieving well-defined ends for the PDA (Figure 1, A). Next, orthogonal cross-sectional contours were created along each hand-picked path to define vessel and stent surfaces (Figure 1, B). For native anatomy, the lumen boundary was followed based on the attenuating contrast in the blood, while in stented segments the metallic structure of the stents was tracked. The contours were defined with an interval of 3 to 5 mm for the aortic and pulmonary vessels, with closer spacing of the contours close to the ends of the PDA (Figure 1, C). The PDA was segmented approximately every 1 mm. For visualization purposes, the cross-sectional surface contours were lofted into a triangulated surface mesh in .stl format (Central Illustration).

**Central Illustration:**
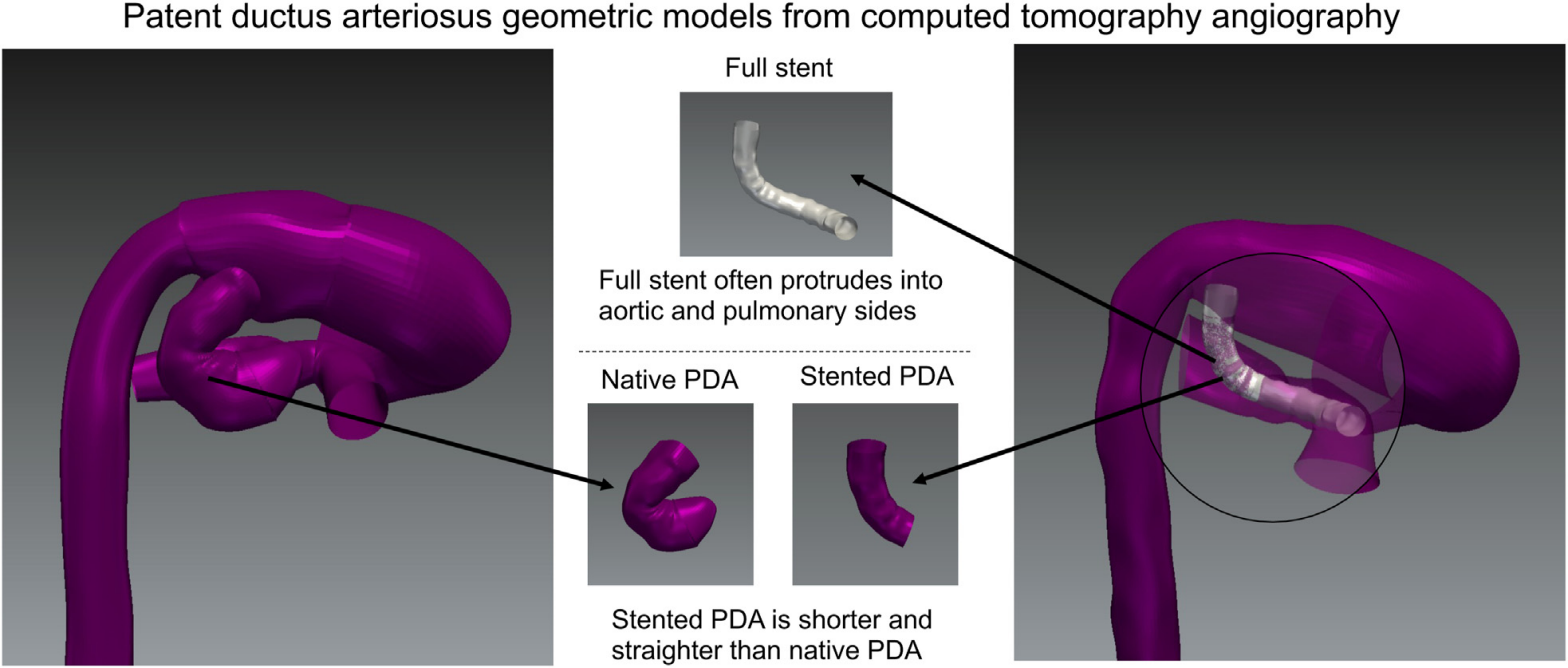
**Patent ductus arteriosus geometric models from computed tomography angiography.** Geometric models of the native aorta, pulmonary arteries, and native- (left) and stented- (right) patent ductus arteriosus (PDA). The full stent is visualized in gray and is often protruding into the aortic and pulmonary sides (middle-top and right). From presenting to poststenting the PDA was significantly straightened and shortened, as visualized (middle-bottom).

### Morphology and deformation quantification

**Figure 2:**
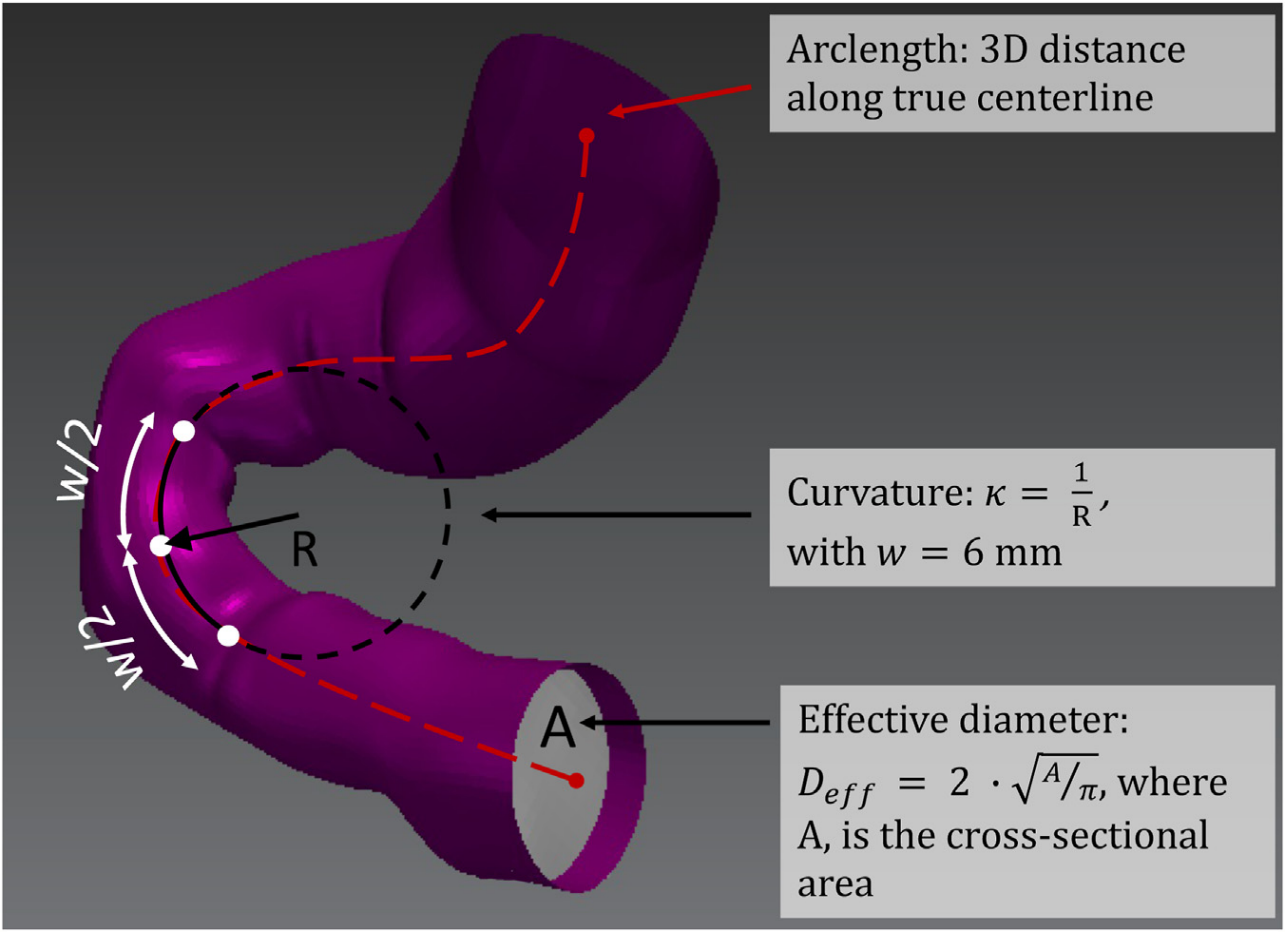
**Schematic definitions of quantified metrics.** Arclength is defined for the vessel/stent centerline, the longitudinal curvature (κ) using a sliding window (w) with curvature defined as the inverse of the fitted circle’s radius (R), and the cross-sectional effective diameter (D_eff_) computed from the cross-sectional area (light gray).

The mathematical centroids of the cross-sectional contours were connected to form true vessel centerlines, which were subsequently smoothed using Fourier smoothing.^15^^,^^16^ These true centerlines were used to compute the arclength and pointwise longitudinal curvature, where curvature was computed using a circle-fitting method that incorporated a sliding window of 6 mm per published recommendations (Figure 2).^15^^,^^17^ Note that the 3 mm (half of the window size) at either end of the PDA or stent were excluded from curvature calculations. Mean curvature was defined as the length-averaged curvature of the entire PDA, and peak curvature was the largest localized curvature value along the length of the PDA. Pointwise effective diameter was extracted from the area of the lumen contours, which were redefined in a Lagrangian coordinate system with additional contours added between existing ones approximately every 0.1 mm using linear interpolation (Figure 2).^17^^,^^18^ Analogous to the longitudinal curvature quantification, effective diameter also utilized a sliding window of 6 mm for measurement noise reduction.

With gated imaging where multiple cardiac frames were captured, longitudinal metrics were calculated for diastole and systole, and these were used to compute deformations. Arclength deformation was computed as percent change from diastole to systole, while the maximum localized change in longitudinal curvature was found from the entire length of interest. Maximum localized change was extracted by first aligning the signals for diastole and systole through dynamic time warping (as the structures of interest may have experienced nonuniform stretching),^15^, ^19^, ^20^ and then calculating the largest localized point-to-point difference between diastole and systole. All stents within a PDA, even multiple overlapping stents, were considered as a single stented segment since individual stents could not be consistently identified in cases of overlap.

### Statistics

The significance of the impact of stenting on PDA morphology (prestenting vs poststenting) was evaluated with 2-tailed, paired *t* tests comparing arclength and curvature. In addition, Spearman’s rank-order correlation was performed to characterize correlation between Qureshi’s tortuosity classification and the impact of stenting on arclength and curvature. The significance of cardiac pulsatility-induced deformation (diastole vs systole) for the native PDA, stented PDA, and PDA stent itself was also evaluated with 2-tailed, paired *t* tests comparing changes in arclength and maximum localized curvature. Statistical significance was defined by a threshold of *P* < .05 and adjusted with Bonferroni-Holm correction for multiple comparisons.^20^

## Results

### Patient cohort

A total of 12 patient datasets were suitable for PDA analyses with n = 11 from Rady Children’s Hospital and n = 1 from Nationwide Children’s Hospital. For each patient, CT image details, relevant diagnoses, sex, age, weight at PDA stenting, and blood pressure are shown in Table 1.

**Table 1:** Patient imaging, relevant diagnoses, demographic characteristics, and blood pressure values.

Patient ID	Scan details, age at scans		Relevant diagnoses	Sex	Age at stent	Weight, kg	Systolic/diastolic BP, mm Hg
	Pre	Post					
Case 1	GR (1 d)	GR (4 mo)	D-TGA, with valvular and subvalvular PS	Male	5 d	3.8	71/32
Case 2	NG (1 d)	GR (2 mo)	PA with intact ventricular septum	Male	2 d	3.3	55/27
Case 3	NG (3 d)	NG (4 mo)	Tetralogy of Fallot with PA	Female	23 d	2.6	88/37
Case 4	GR (24 d)	GR (4 mo)	DORV, L-TGA, large outlet VSD, PA	Male	1 mo	3.0	60/30
Case 5	NG (2 mo)	NG (6 mo)	DORV, D-TGA, PA	Male	5 mo	1.6	75/30
Case 6	GR (6 mo)	GR (7 mo)	D-TGA with valvular and subvalvular PS	Male	6 mo	8.5	58/32
Case 7	GR (3 d)	GR (4 mo)	DORV, PA, VSD	Female	17 d	3.8	72/22
Case 8	GR (0 d)	N/A	Tricuspid atresia, L-TGA, large VSD, PS	Female	12 d	3.1	61/24
Case 9	GR (3 d)	N/A	L-TGA, VSD, PA	Female	15 d	3.4	81/25
Case 10	GR (3 d)	N/A	DILV, D-TGA, PA	Male	18 d	3.5	70/33
Case 11	GR (2 d)	N/A	DILV, PA	Male	4 d	3.9	53/27
Case 12	GD (3 d)	GD (9 mo)	D-TGA, large VSD, PA	Male	10 d	4.1	78/39

BP, blood pressure at the time of stenting; DILV, double inlet left ventricle; DORV, double outlet right ventricle; D-TGA, dextro-transposition of the great arteries; GD, gated diastole only, GR, gated cardiac-resolved; L-TGA, levo-transposition of the great arteries; N/A, not available; NG, nongated; PA, pulmonary atresia; PDA, patent ductus arteriosus; PS, pulmonary stenosis; VSD, ventricular septal defect.

### Native and stented PDA morphology and impact of stenting

PDA stenting details, diastolic PDA arclength and curvature before and after stenting, impact of stenting on PDA arclength and mean curvature, PDA tortuosity classification,^21^ and diastolic native PDA diameter are shown in Table 2.

**Table 2:** PDA stent details, diastolic PDA arclength and curvature before and after stenting, PDA tortuosity classification, and diastolic native PDA diameter.

Patient ID	Stent design diameter × length, mm PTA pressure(s)	Diastolic arclength, mm Native stented %change	Diastolic mean curvature, mm^-1^ Native stented %change	Diastolic peak curvature, mm^-1^ Native stented %change	PDA tortuosity class ^21^	Diastolic native diameter, mm Mean min-max range
Case 1	Resolute Onyx 3.5 × 22 12 atm	15.2 16.4 +8%	0.25 0.18 -29%	0.42 0.25 -42%	II	2.8 2.7-3.0 0.3
Case 2	Resolute Onyx 3.5 × 26 18 atm	24.2 22.1 -9%	0.17 0.07 -62%	0.33 0.12 -64%	II	5.1 4.8-5.8 1.0
Case 3	Resolute Integrity (× 2) 3.5 × 18, 4.0 × 15 20, 20 atm	18.8 12.2 -35%	0.27 0.06 -80%	0.51 0.07 -87%	III	3.1 2.7-3.8 1.1
Case 4	Resolute Onyx 3.5 × 18 12 atm	10.5 7.1 -32%	0.24 0.06 -77%	0.28 0.06 -78%	I	4.2 3.7-4.6 1.0
Case 5	Resolute Onyx 4.0 × 15 18 atm	29.2 18.8 -36%	0.20 0.14 -30%	0.50 0.20 -60%	II	3.6 2.9-4.6 1.7
Case 6	Resolute Onyx 3.0 × 15 15 atm	15.7 9.3 -41%	0.35 0.09 -74%	0.55 0.12 -77%	II	2.1 1.8-2.6 0.8
Case 7	Resolute Onyx 3.5 × 22 14 atm	17.1 10.1 -41%	0.26 0.14 -47%	0.33 0.15 -55%	II	6.0 5.7-6.3 0.6
Case 8	Resolute Onyx 3.5 × 18 14 atm	16.3	0.14	0.19	I	4.8 4.6-5.3 0.8
Case 9	Resolute Onyx (× 3) 3.0 × 30, 3.5 × 18, 3.5 × 8 12, 8, 12 atm	17.4	0.30	0.57	III	3.7 3.4-4.1 0.6
Case 10	Resolute Onyx 3.5 × 22 16 atm	15.9	0.24	0.43	III	4.5 4.2-4.8 0.6
Case 11	Resolute Onyx 3.5 × 22 18 atm	17.6	0.22	0.32	II	6.0 5.7-6.2 0.5
Case 12	Resolute Integrity 4.0 × 18 12 atm	13.8 11.2 -19%	0.25 0.05 -79%	0.47 0.10 -79%	II	4.9 4.6-5.4 0.8

Avg ± SD		18.1 ± 6.0	0.25 ± 0.05	0.43 ± 0.10	NA	Range =
(Paired native vs stented)		13.4 ± 5.2	0.10 ± 0.05	0.13 ± 0.06		0.8 ± 0.4
		-26 ± 18%	-60 ± 21%	-68 ± 15%		
*P* value		.004	< .001	< .001		

T tests show the significance of the impact of stenting from native to stented PDA.

Avg, average; mean, PDA length-averaged effective diameter; min-max, minimum and maximum effective diameter along the PDA length; NA, not applicable; PDA, patent ductus arteriosus; PTA, percutaneous transluminal angioplasty.

**Figure 3:**
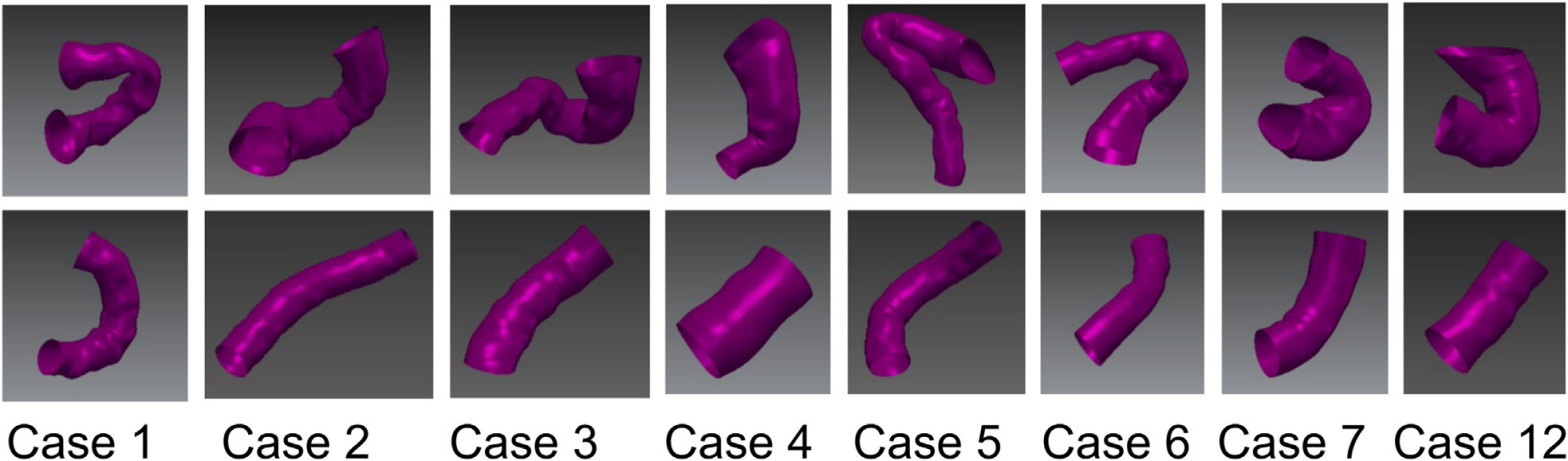
**In 8 patients, 3-dimensional models of native (top row) and stented (bottom row) patent ductus arteriosus were analyzed.** In the left 4 columns, the surface models shown are from nongated (diastole-weighted) images, while in the right 4 columns, the surface models are the diastole models from gated images.

Diastolic images were acquired before and after stenting for n = 8 patients, for which PDA morphology were modeled (Figure 3), and paired differences between prestenting and poststenting were computed (Table 2). The PDA shortened significantly by 26 ± 18% (*P* = .004, maximum of 41%) and decreased in mean curvature by 60 ± 21% (*P* < .001, maximum of 80%) and peak curvature by 68 ± 15% (*P* < .001, maximum of 87%), from prestenting to poststenting. There were no correlations between tortuosity classification and arclength shortening (R = −0.109 vs critical value R = 0.738) and decrease in mean and peak curvatures (R = −0.218 and −0.218 vs critical value R = 0.738). Mean, minimum, maximum, and range of PDA diameter are shown for each patient, yielding a cohort average range of 0.8 ± 0.4 mm (Table 2).

### Cardiac pulsatility-induced deformation of native PDA, stented PDA, and PDA stent itself

Cardiac-resolved imaging was acquired for n = 8 patients before PDA stenting and n = 5 patients after stenting. Arclength and maximum localized curvature changes for the native PDA, stented PDA, and the PDA stent itself, due to cardiac pulsatility (from diastole to systole), are shown in Table 3. Peak PDA stent curvature is also shown (peak curvatures for the native and stented PDA are already shown in Table 2). There were no significant pulsatile deformations in the native PDA, stented PDA, or PDA stent itself.

**Table 3:** Cardiac pulsatility-induced deformations (from diastole to systole) of the PDA before and after stenting, and of the PDA stent itself.

Patient ID	Native PDA arclength change, %	Max native PDA curvature change, mm^-1^	Stented PDA arclength change, %	Max stented PDA curvature change, mm^-1^	PDA stent arclength change, %	Max PDA stent curvature change, mm^-1^	Peak PDA stent diastolic curvature, mm^-1^
Case 1	3.8	-0.17	-1.1	0.02	4.3	-0.03	0.25
Case 2	–	–	-0.1	0.01	2.1	0.02	0.12
Case 4	14.4	-0.08	8.2	0.03	-1.0	0.02	0.13
Case 6	-2.3	-0.10	17.0	0.08	3.1	0.06	0.16
Case 7	-7.3	-0.13	-3.0	-0.01	-0.9	0.01	0.12
Case 8	10.0	-0.10	–	–	–	–	–
Case 9	4.9	0.09	–	–	–	–	–
Case 10	-2.8	-0.09	–	–	–	–	–
Case 11	5.5	0.04	–	–	–	–	–

Avg ± SD	3.3 ± 7.1	-0.07 ± 0.09	4.2 ± 8.3	0.03 ± 0.03	1.5 ± 2.4	0.01 ± 0.03	0.15 ± 0.06
*P* value	.237	.065	.323	.140	.220	.398	NA

T tests show the significance of the impact of cardiac pulsatility from diastole to systole.

Avg, average; NA, not applicable; PDA, patent ductus arteriosus.

## Discussion

### Native and stented PDA morphology and impact of stenting

The significant shortening (average = 26 ± 18%) and straightening (mean = 60 ± 21%, peak = 68 ± 15%) of the PDA from prestenting to poststenting provide critical information for interventionalists. First, these quantitative data confirm physicians’ beliefs and anecdotal evidence that have led to the use of stents shorter than the native PDA length. Without this adjustment, the stent can encroach upon the aortic and pulmonary artery lumens (Central Illustration), potentially causing hemodynamic disturbances. However, if insufficient stent length is used, part of the PDA may be exposed, introducing the risk of vessel narrowing at either end of the stent and eliminating the communication between systemic and pulmonary circuits. These data provide an initial guide to PDA stent length selection, which may be refined in the future based on native PDA tortuosity. The dramatic straightening of the PDA is an indication of the magnitude of bending stiffness of the implanted stents and is consistent with the straightening effect observed in coronary arteries after stenting.^22^ Additionally, this informs us that a more conformable stent design may be favorable in the PDA, with the benefits of being able to follow the native morphology better, improving stent sizing and stent-to-native vessel transitions.

### Cardiac pulsatility-induced deformation of native PDA, stented PDA, and PDA stent itself

While pulsatile deformations in the native PDA were not statistically significant, logical trends were observed. From diastole to systole, there was a nonsignificant increase in arclength and nonsignificant straightening at the location of maximum bending (Table 3). These changes make sense because a prebent, unconstrained, flexible tube would be expected to lengthen and straighten with higher pressurization. There are large standard deviations for the cohort data due to the underlying high variation in morphology and deformation between patients.

There were also no significant pulsatile deformations found for the stented PDA; however, certain trends are worth noting. While both the native and stented PDA trended longer during systole, the native PDA trended more straight during systole while the stented PDA trended more curved during systole. This is potentially due to the aorta and pulmonary artery translating closer to each other during the high-pressure state of systole and forcing the stented PDA to bend. This phenomenon could also be due to the ends of the stent being pinned to the vessel wall, forcing it to bow out during higher pressurization. Both the arclength and maximum curvature change range from negative to positive across the patient population, which indicates that deformations are not purely driven by pressure changes in the PDA, but also by the movement of the aorta and pulmonary artery that tether the PDA on either side. This altogether means that PDA stents need to accommodate highly dynamic environments in worst-case scenarios, ie, be highly flexible, and not only for average cases.

**Figure 4:**
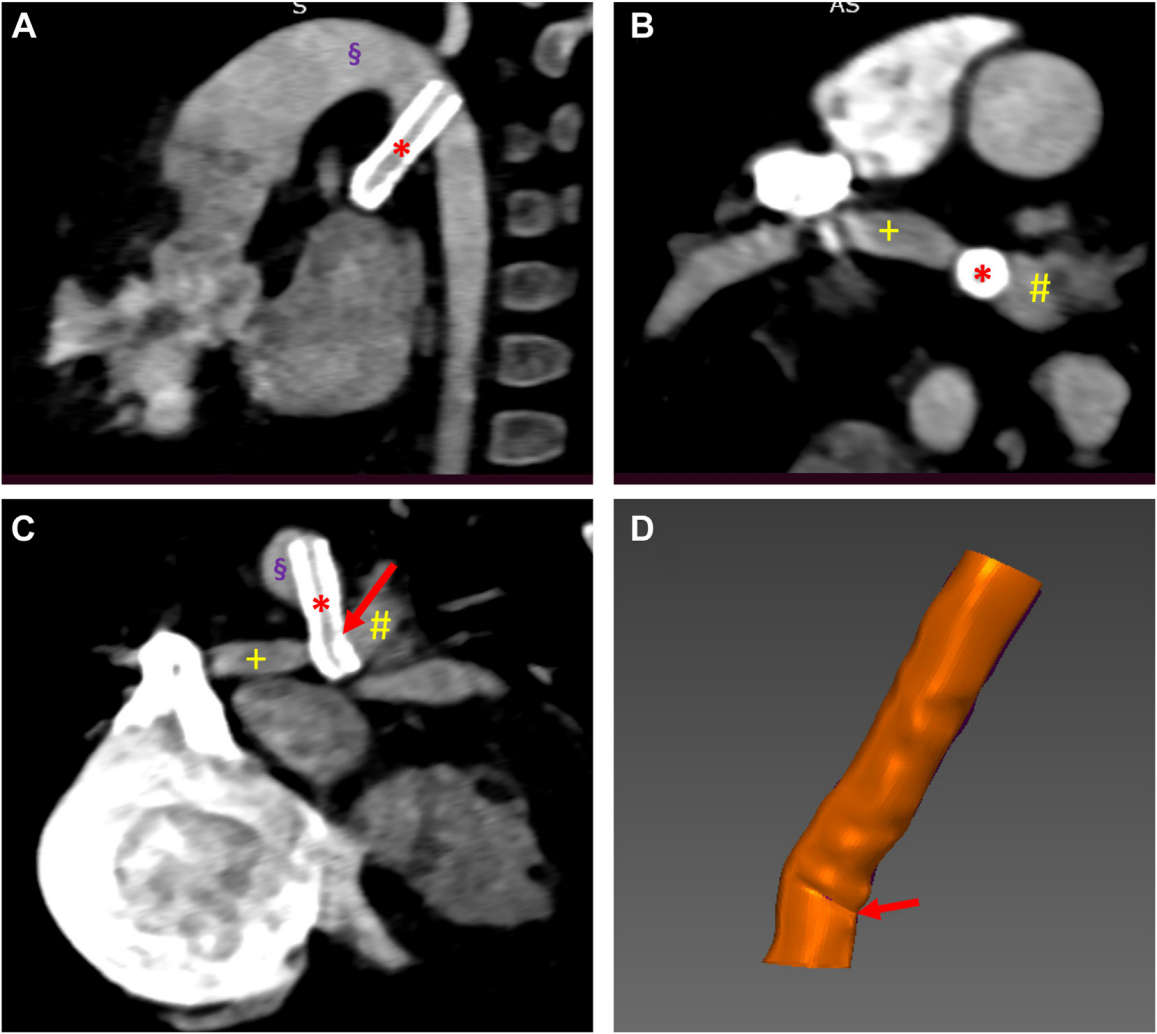
**Images from double-oblique planes cutting through** (**A-C**) **the stented PDA and** (**D**) **3-dimensional stent model for case 4 where the stent abuts against the vessel wall of the left pulmonary artery (yellow #), leading to stent kinking (red arrow) in the lumen of the pulmonary artery.** The right pulmonary artery (yellow +), aorta (purple §), and stented PDA (red ∗) are also marked.

On average, the PDA stent itself exhibited insignificant and very low levels of pulsatile length and bending deformations, indicating that PDA stent fractures, at least for coronary stent designs, may not be caused by dynamic bending of the PDA vessel. However, for case 4, the diastolic peak curvature of the Resolute Onyx (Medtronic) stent was 0.12 mm^-1^, which is 2 times the peak curvature of the stented PDA (0.06 mm^-1^, Table 2). This reveals that the PDA stent may experience its peak curvature outside of the PDA, within the aorta or pulmonary artery where the stent is not supported by the PDA, as exemplified in patient case 4 (Figure 4). Note that the kink most likely occurred during stent deployment, but this kink superposed with pulsatile deformation can lead to accelerated fatigue damage due to the combination of high mean stress (from abutting against the vessel wall) and the alternating stress (from the pulsatile motion).

### Limitations and future work

While this study presents novel biomechanical data on the PDA before and after stenting, it included only 2 stent designs produced by a single manufacturer and was limited by a small, heterogeneous (eg, age, sex, weight, disease etiology) patient cohort. The cohort exhibited large standard deviations in data, underlain by high variation in morphology and deformation between patients. Most patients were treated with single stents; however, 2 patients received multiple overlapping stents; due to limited radiopacity of the stents, overlapped regions could not be resolved, so overlapping stents were treated as a single stented region. Since the inclusion of patients was retrospective, the amount of time that elapsed between stenting and poststent CT imaging was variable, which resulted in varying amounts of growth and remodeling after intervention. Another limitation that follows with the retrospective data collection is the underlying bias introduced by only being able to include those patients with appropriate CT data available. In addition, the subcohort of patients who received both prestent and poststent imaging was even smaller due to the urgent and complex nature of treatment. On this note, the poststent indication for getting a CT was for surgical planning of next-stage palliation, which further introduced cohort bias.

Given the results of this pilot study, prospective studies with larger patient cohorts are planned to address these limitations. The aim of this follow-up study is, in addition to including more patients, to leverage machine learning methods to predict poststent morphology based on native features such as arclength, curvature, and pulsatility.

## Conclusions

This retrospective clinical study quantifies in vivo morphology and cardiac pulsatility-induced axial and bending deformations of PDA before and after stenting in patients with cyanotic congenital heart disease. Stenting causes the PDA to shorten by an average of 26 ± 18%, and if the stent extends beyond the span of the PDA, it can encroach upon the flow lumen in the aorta and/or pulmonary artery and experience a kink, potentially contributing to mechanical fatigue. The native PDA exhibits a large lengthwise diameter range, making lengthwise quantification of diameter helpful for preoperative planning. After stenting, the PDA exhibits decreased longitudinal curvature by 60% to 70%, and a near absence of pulsatile bending. This quantitative understanding of how stenting impacts PDA morphology could be critical for device selection in current clinical practice, and the pulsatile deformations of PDA stents could be used in the evaluation of current devices, as well as the development of future devices specific to congenital heart disease in neonates.
