# Supplementary material for: Impact of Stenting on PDA Length, Curvature, and Pulsatile Deformations Based on CT Assessment
Source: J Soc Cardiovasc Angiogr Interv. 2023 Sep 16;2(6Part A):101134. doi: 10.1016/j.jscai.2023.101134 (PMC11307392; doi:10.1016/j.jscai.2023.101134)
Supplement: Supplementary Data [file mmc1.docx]

**Social Media Abstract**
CTA of newborns with #CHD pre and post #PDA stenting to quantify changes to morphology and deformation. PDAs shortened and straightened after stenting, informing stent sizing and product development.
